# Longitudinal Transition Between Regular and Special Education in Autistic Children: Predictors and Policy Effects

**DOI:** 10.1007/s10803-024-06369-4

**Published:** 2024-05-20

**Authors:** Chantal van den Helder, Rachel Plak, Martijn Meeter, Sander Begeer

**Affiliations:** 1 Department of Clinical, Neuro and Developmental Psychology, Faculty of Behavioural and Movement Sciences, Vrije Universiteit Amsterdam, Amsterdam, The Netherlands; 2 Institute of Education and Child Studies, Faculty of Social and Behavioural Sciences, Leiden University, Leiden, The Netherlands; 3 LEARN! Research Institute, Vrije Universiteit Amsterdam, Amsterdam, The Netherlands

**Keywords:** Autism, Children, School placement, Inclusive education, Policy, Longitudinal

## Abstract

Inclusive education policies stimulate children with special educational needs, including autism, to attend regular education. We aimed to explore change over time in school placement and transitions of autistic children since the introduction of an inclusive education policy in the Netherlands (2014) and to examine the role of individual child characteristics. This study used longitudinal data from 2013 to 2021 on autistic children (N = 1463, aged 5–16 years). We expected an increase in regular school placements and transitions to regular schools. Surprisingly, the proportion of children with autism in regular schools slightly decreased. Special school placement was more likely for boys and autistic children with lower intelligence scores, co-occurring conditions or behavioral and peer relation problems. Younger autistic children and those with lower intelligence scores more often transferred from a regular to a special school. The opposite transition was more common in older autistic children and those with higher intelligence scores. The likelihood of special-to-regular transitions did not change, but regular-to-special transitions were more prevalent during the years just after the policy change than in later years. The inclusive policy had little impact on the school placement, and at most led to a delayed drop in referrals to special schools.

**Supplementary Information:**

The online version contains supplementary material available at 10.1007/s10803-024-06369-4.

## Introduction

Many countries have schools caring for and catering to children with special educational needs (SEN). While such schools offer support that may be lacking in regular schools, they also segregate children with SEN from local children. Moreover, the existence of special schools leads to a dichotomy between children with and without special needs, where a continuum from less to more severe special needs would be more appropriate Lindsay et al. (2020). Educational policies have been implemented in various countries to promote education of children with SEN, including autism, in regular schools (Van Kessel et al., 2021). However, for autistic children, it is unknown whether and for whom such inclusive policies have impacted on regular or special school attendance. In this study we explore differences in the proportions and characteristic of children with autism in special and regular educational settings since the latest implementation of inclusive education policy in the Netherlands over the period from 2013 to 2021.

The implementation of national policies, such as the inclusive policy, has great impact on the accessibility of education for children with autism (Lord et al., 2022). For example, in the USA, the proportion of autistic children in regular classes increased in the years following consecutive introduction inclusive policies in 1990, 1997 and 2004 and the proportion of children with autism placed in a special school decreased within each of the three reform periods (De Bruin, 2019). This was also found in Israel, where the placement of autistic children in regular schools increased and that in special schools decreased (Gindi, 2020). In contrast, in Australia, after the introduction of inclusive policy in 2005, there has been a shift towards placement in special educational settings (De Bruin, 2019).

The educational system in the Netherlands comprises three main types of schools: regular schools, special primary schools for moderate support (designed mainly for children with SEN having less severe learning impairments) and special schools for children with special educational needs requiring extensive support. In 2014, the introduction of the Appropriate Education Act (AEA) facilitated a collaborative framework for these schools organizing them in “regional alliances” to provide customized support within a fixed budget, for children with SEN. Each school autonomously determines the range of support arrangements it can offer. The criteria for admission to special schools are set by these regional alliances. When evaluating a child for placement, the regular school consults the parents and when feasible the child, to determine whether the placement is appropriate for the child. If the school determines that it cannot adequality support the child and placement in a special school is preferable, the regional alliance decides whether the criteria for admittance are met (European Agency for Special Needs and Inclusive Education, 2020; Steunpunt Passend Onderwijs, 2013).

The proportion of children with SEN placed in special schools and classes varies within European countries where inclusive education is applied (Ramberg et al., 2020). This variation reflects heterogeneity in inclusive education policies across countries (Watkins et al., 2021). Prior to implementing inclusive policies in the Netherlands, the proportion of SEN students placed in a special school was high (De Boer, 2020; Ledoux et al., 2020). Placement of children with SEN in special educational settings increased in the first years after the introduction and then declined to pre-introduction levels (Inspectie van het Onderwijs, 2021; Ministerie van Onderwijs Cultuur en Wetenschap, 2019). However, studies on the impact of inclusive policy on autistic children are lacking.

The AEA focuses on the support a child needs to attend education, given its characteristics. Therefore, it is important to explore what child characteristics are predictive of placements in special schools. For autistic children, attending regular rather than special education primarily depends on cognitive skills and symptom severity (White et al., 2007; Yianni-Coudurier et al., 2008). White et al. (2007) found that autistic children who remained in special education throughout middle school had lower IQ scores than their peers who started in regular education and remained there. No association with IQ scores was found for the few children with autism who made a transition from a special to a regular class. Autistic children in special school settings typically display more externalizing behavioral problems and less adaptive and social skills compared to their peers in regular settings (Lauderdale-Littin et al., 2013; Sansosti & Sansosti, 2012; Towle et al., 2018; White et al., 2007; Yianni-Coudurier et al., 2008). Disruptive problem behaviors were related to regular to special transitions (Rattaz et al., 2020; Sansosti & Sansosti, 2012; Yianni-Coudurier et al., 2008), higher socialization scores with special to regular transitions (White et al., 2009). Not many studies examined the specific behavioral symptoms in relation to the school placement and transition of children with autism.

Variation in co- occurring conditions can affect autistic children’s access to education (Lord et al., 2022; Morningstar et al., 2017). Social anxiety disorder, Attention Deficit/ Hyperactivity Disorder and Oppositional Defiant Disorder are the most common co-occurring conditions in autistic children (Lord et al., 2022; Salazar et al., 2015; Simonoff et al., 2008). In the study of Rattaz et al. (2020) symptoms of anxiety were more common in children with autism placed in special classes than in their autistic peers who were placed in regular classes. However, the association between the nature and severity of co- occurring conditions and school placement and transitions between regular and special schools of autistic children is unclear.

Gender differences in school placement and transitions among autistic children are also under-examined. There is a risk of under-recognizing signs of autism for autistic girls, which can negatively affect their education (Halsall et al., 2021; Lord et al., 2022; Wood-Downie et al., 2020). While various studies did not identify gender differences in the school placement (May et al., 2014; White et al., 2007), girls with autism more often followed regular education compared to boys with autism (Yianni-Coudurier et al., 2008). However, these findings were based on low number of girls.

Current findings do not identify a clear pattern of school placements and transitions among children with autism in different age categories. In the study of Towle et al. (2018) in the USA, most transitions from special to regular classes took place in the period of preschool and kindergarten. From kindergarten to 8th grade, in the USA, children with autism mostly remained in the school placement where they started (White et al., 2007). A longitudinal study in Irael among autistic children from 1st to 12th grade showed that the change from 6th to 7th grade was the key period for a transition to a special school (Gindi, 2020). Furthermore, a few studies showed a negative relation between socioeconomic status (SES) and the school placement and transition of children with autism (Gindi, 2020; Yianni-Coudurier et al., 2008). On the other hand, Lauderdale-Littin et al. (2013) showed, that autistic child’s family income was positive related to the likelihood of placement in a special school.

In the current study, we aim to examine whether (1) the proportion of autistic children attending education in a special school changed over time in the period from 2013 to 2021, (2) transitions between regular and special schools of children with autism changed in frequency since the introduction of the inclusive education policy in the Netherlands, and (3) child characteristics are related to school placement and transitions.

## Methods

### Design

This study is a longitudinal cohort study using data collected through the Netherlands Autism Register (NAR). The NAR was established by the Dutch Association for Autism (Nederlandse Vereniging voor Autisme; NVA) in collaboration with the Vrije Universiteit Amsterdam (VU), for the purpose of identifying the differences and similarities of people with autism and to help autistic people to protect their interest and improve their quality of live (https://www.nederlandsautismeregister.nl/english/).

### Participants

Participants included 1463, mostly high educated (> 69%) adult caregivers (parents or legal guardians), of autistic children (see Table 1) who reported on their child by filling in questionnaires from the Netherlands Autism Register.

**Table 1:** Descriptive statistics per wave

Wave	*N*	Girls (%)	Age	
			*M*	SD
2013	847	17.83	11.27	3.83
2015	555	19.27	11.82	2.60
2016	331	18.75	12.33	2.62
2017	399	19.03	12.00	2.81
2018	379	19.80	11.93	2.73
2019	332	21.90	11.96	2.81
2020	312	22.29	11.62	2.88
2021	230	24.68	12.05	2.70
Total	3385	23.04	11.78	2.77

All children had received a confirmed autism spectrum diagnosis according to DSM-IV and DSM-V (American Psychiatric Association, 1994, 2013) criteria by a qualified clinician unaffiliated to this study. Caregivers reported the diagnosis and follow-up questions ensured that the diagnosis was given by a qualified clinician at a professional location.

### Measures

#### School Placement

Placement was measured using the current type of education attendance that caregivers reported for their child. In the Netherlands, two kinds of special schools exist, with names that translate to *special education* (special schools as they exist in many countries), and *special primary education*. In the latter, the regular curriculum is taught but at a slower pace and with extra support. We grouped these two kinds as special schools and contrasted it with regular schools. However, it is also possible to contrast *special education* with *regular* + *special primary education*. Results of those analyses are reported in the supplemental material.

#### Transition

Transition in the child’s type of education attendance reported from one wave to the next. Transition was categorized in three categories: 0 = no change, 1 = transition to more regular (from special school to a special primary or regular school or from a special primary school to a regular primary school), 2 = transition to more special (from a regular to special primary or special school, or from special primary school to a special school).

#### Gender

Participants could choose between male, female or other.

#### Family Socioeconomic Status

Parental educational level was used to measure the family socioeconomic status. It was categorized into seven categories from low (primary school) to high (university), using the highest of the two parents.

#### Intelligence

Participants were asked to choose their child’s most recent IQ test score from seven categories, these categories were then recoded in order:1 = below 40, 2 = 41 to 55, 3 = 56 to 70, 4 = 71 to 85, 5 = 86 to 115, 6 = 116 to 130, 7 = above 130 and entered in the analysis as an interval variable coded from 1 to 7.

#### Autistic Traits

The Autism-Spectrum-Quotient (AQ)- short (Hoekstra et al., 2011) was administered at the time of enrollment in NAR. The AQ-short includes 28 items that assess a fascination for numbers/patterns (5 items) and social behavioral difficulties (23 items). The social behavioral factor includes social skills (7 items), routine (4 items), switching (4 items) and imagination (8 items). At the NAR registration, participants were invited to respond to a four-point Likert scale from 1 = totally agree to 4 = totally disagree. Higher total scores represented higher autism severity and impaired social skills. The AQ- short total scores has shown a good internal consistency with a Cronbach’s alpha between 0.77 and 0.86. The AQ- short highly correlates with the full scale AQ (*r* between 0.93 and 0.95) (Hoekstra et al., 2011).

#### Behavioral Indicators

The Strengths and Difficulties Questionnaire (Goodman, 1997, 2001) was used to assess behavioral indicators. The SDQ is a brief behavioral screening questionnaire (25 items) describing the positive and negative attributes rated on a three- point Likert scale. The SDQ consists of five subscales assessing emotional symptoms, conduct problems, hyperactivity- inattention, peer relationship problems and pro-social behavior each measured with five items respectively. The reliability and validity of parent report SDQ were judged to be satisfying (*α* = 0.73) (Goodman, 2001). The SDQ was administered in 2017 and 2018; the most recent score was used.

#### Co-occurring Conditions

We coded whether parents reported one or more diagnosed co- occurring conditions by a qualified clinician or not.

#### Age

Age of the child was reported by the participants in the baseline questionnaire.

#### Year

Year was coded as the number of the wave.

#### Inclusion Policy

We created a dichotomous policy variable coding whether the wave was before the implementation of the inclusion policy (2013) or after it (2015 and later waves).

#### Missing Values

Over the time, there was a decrease in sample size (872 participants in 2013 versus 243 in 2022). This resulted in missing datapoints for autistic traits (AQ-short), behavioral indicators (SDQ) and family socioeconomic status. The number of measurements for the predictor variables in each wave from 2013 to 2021 is presented in table S1 in the supplemental.

### Procedure

All data for this study were obtained from the NAR, a longitudinal cohort database (see https://www.nederlandsautismeregister.nl/english/). Upon entering the NAR, participants receive a link to fill in an online baseline questionnaire, followed by yearly invitations to fill in an online questionnaire. Every year, participants can decide whether they want to participate. For this study, eight waves were used covering the years from 2013 to 2021, with the exception of 2014 for which there was no data collection. Parent reports were used on children aged 5 to 16 years, the ages at which children visit primary or secondary school and caregivers are legally allowed to report on their child.

### Statistical Analysis

Placements in regular versus special school at wave 2013 to wave 2021 were subjected to a hierarchical logistic analysis with two levels: wave, nested within the level of the child. Included predictors at the level of the wave were age of the child, and year of the wave (year since 2013, quadratic trend of year, and implementation of the inclusion policy (2013) or after it (2015 +). We centered year and its quadratic trend. Predictors at the level of the child were gender, socioeconomic status, intelligence, autistic traits, behavioral indicators and co- occurring conditions.

We used a hierarchical logistic regression, entering first the variables at the level of the child, then age, then the policy variable, and finally the linear and quadratic trend of year. We examined for multicollinearity, using a VIF statistic lower than 2.5 as criterion.

To further explore individual predictors of placement into special schools, we analyzed the subscales of the Strengths and Difficulties Questionnaire.

We next turned to analysing transitions between regular and special schools. The placement analysis has as drawback that the sample varies from wave to wave. Differences in the distribution of placements can therefore also be explained as resulting from differences in samples. In our transition analysis, we focused on children whose parents participated in two subsequent waves, reducing the risk that a shift in sample characteristics could result in spurious findings, since new participants or participants who stop in between waves would not be part of the analysis.

Due to limited observed transitions, we decided against the analysis planned in the preregistration. Instead, we analyzed whether children who were in regular schools, would remain there or transitioned to special schools in the next wave, and then whether children who were in special schools in one wave would transition to a regular school in the next wave. This dependent variable was subjected to a hierarchical multi- level logistic analysis with as levels wave, and child. We first entered child-level variables (the same as in the placement analysis: gender, socioeconomic status, intelligence, autistic traits, behavioral indicators and co- occurring conditions). Then we entered age at the moment of the transition, then year of wave and its quadratic trend.

## Results

**Fig. 1:**
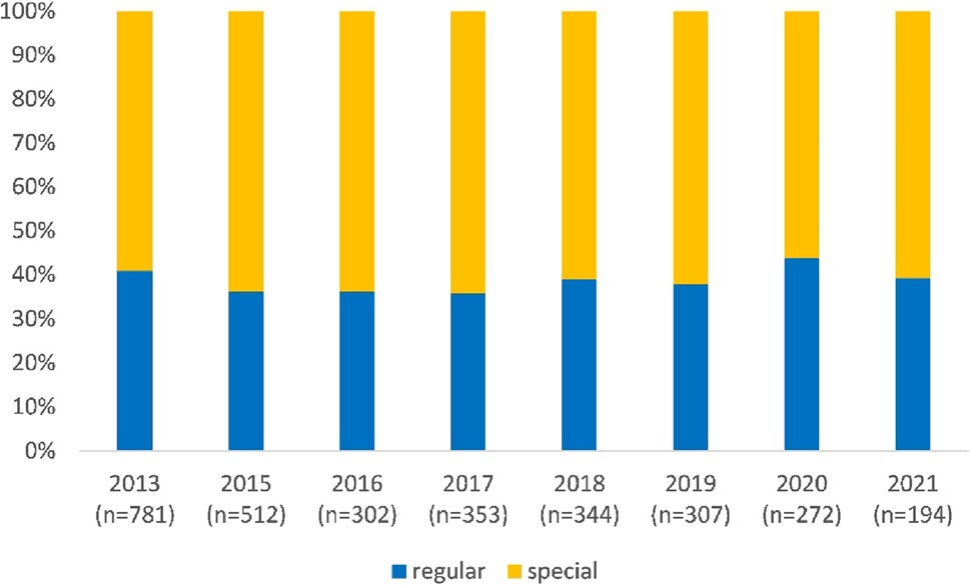
Number of children in regular and special school in each wave

Figure 1 shows the number of children in regular and special schools for each wave from 2013 to 2021.

We first set out to assess the relation between the proportion of children in regular vs special schools, and variables at the level of the child and of the wave. Some variables had one or more significant correlation coefficients, nonetheless, the Variation Inflation Factor (VIF) statistics for the variables were all lower than 2.5, see Table 2.

**Table 2:** Correlation coefficients between the independent variables

Predictors	SES	IQ	Autistic traits	Behav indicators	Co- occurring conditions	Age of child	Inclusive education policy	Year	Quadratic trend of year
Gender^a^	.00	− .02	− .08**	.10**	.02	.01	.04*	.05**	.02
SES		.09**	− .12**	− .22**	− .15**	− .04*	.04*	.09^**^	.06**
Intelligence (IQ)			− .04	− .23**	− .04*	.06**	− .06**	− .04*	.04*
Autistic traits				.32**	.08**	− .06**	.02	− .01	− .04
Behav indicators					.32**	− .10**	.02	.03	− .00
Co- occurring conditions						.03	− .01	− .01	.01
Age of child							.11**	.06**	− .04*
Inclusive Education Policy								.67**	− .22**
Year									.42**

*p < 0.05, **p < 0.01

^a^Gender coded as 1: male; 2: female

For the placement analysis, a hierarchical logistic regression analysis was performed to assess the effects of the predictors on the likelihood of child’s placement in a special school versus regular school at a specific wave. In model 1 we introduced only the child level variables as predictors. The likelihood of a child being placed in a special school was higher for boys, for children with lower intelligence, co- occurring conditions and higher scores on behavioral indicators (*p* < 0.05). Socioeconomic status and autistic traits were not related to the likelihood of placement in a special school. In model 2 we added age of the child and then in model 3 inclusive education policy as predictors to the model. Both were not related to the likelihood of placement in a special school. In model 4, we introduced year (linear and quadratic trend) as predictors; neither were related to the likelihood of placement in a special school. The findings for other variables did not change, see Table 3.

**Table 3:** Results for the placement in a special school

Predictors	Model 1		Model 2		Model 3		Model 4		Model 5	
	Estimate	SE	Estimate	SE	Estimate	SE	Estimate	SE	Estimate	SE
Gender^a^	− .62***	.17	− .62***	.17	− .63***	.17	− .63***	.17	− .48**	.18
Socioeconomic status	− .07	.04	− .07	.04	− .07	.04	− .07	.04	− .06	.04
Intelligence	− .47***	.08	− .47***	.08	− .47***	.08	− .48***	.08	− .46***	.08
Autistic traits	− .00	.01	− .00	.01	− .00	.01	− .00	.01	− .00	.01
Behavioral indicators	.03*	.01	.03*	.01	.03*	.01	.03*	.01		
Emotional problems									− .06*	.03
Behavioural problems									.09*	.04
Hyperactivity/inattention									.05	.03
Peer problems									.09**	.03
Co-occurring conditions	.74***	.14	.74***	.14	.74***	.14	.74***	.14	.69***	.15
Age of the child			.02	.03	.00	.03	.03	.03	.02	.03
Inclusive education policy					.17	.21	.52	.31	.54	.32
Year							− .11	.06	− .11	.06
Quadratic trend of year							.02	.02	.02	.02
Constant	2.70***	.79	2.52**	.85	2.52**	.85	1.93*	.92	1.37	.97
*Chi*^*2*^	(*Df* = 6) 112.87**		(*Df* = 7) 113.22***		(*Df* = 8) 113.92***		(*Df* = 10) 117.21***		*(Df* = 13) 131.62***	

*p < 0.05, **p < 0.01, ***p < 0.001

^a^Gender coded as 1: male; 2: female

To assess the individual contribution of the various behavioral indicators, in Model 5 we replaced SDQ scores with the following behavioral indicator subscores: emotional problems, behavioral problems, hyperactivity/ inattention and peer problems. As shown in Table 3, behavioral problems and peer problems were both associated with higher likelihood of placement in a special school in each model (*p* < 0.05). Emotional problems (B = − 0.06, SE = . 03*,* *p* = 0.045) were, paradoxically, associated with a higher likelihood of placement in a regular school. In none of the models hyperactivity/inattention was related to the likelihood of placement in a special school.

Due to missing values for autistic traits and behavioral indicators, there was a loss of datapoints in the above reported analysis. To assess the contribution of the inclusive education policy and year in the overall sample, we repeated the analysis without these variables. This increased the sample from 1085 to 2367. In each model the inclusive education policy (B = 0.44, SE = 0.21*,* *p* = 0.038) was associated with an increase in likelihood of placement in special education. Year (B = − 0.04, SE = 0.04*,* *p* = 0.286), however, remained a non-significant predictor for the likelihood of placement in special education.

Table 4 shows the number of transitions occurring between two waves, between regular and special education. Only 5% of the children have been transitioned between school settings more than once, all from regular to special back to a regular school.

**Table 4:** Transitions per year

Transitions	2013–2015	2015–2016	2016–2017	2017–2018	2018–2019	2019–2020	2020–2021
No transition	329	273	237	218	224	152	170
Regular school	123	98	77	81	79	59	64
Special school	206	175	160	137	145	93	106
Transition regular to special	36	8	6	13	7	2	6
Transition special to regular	13	4	6	6	13	3	7
*N*	378	285	249	237	244	157	183

We first analyzed the likelihood of a transition from regular to special schools, versus staying within regular schools, and then the likelihood of a reverse transition. The requirement that a child be in the sample of two adjacent waves, together with the high number of missing values for autistic traits and behavioral indicators, led to small samples. We therefore decided to perform the transition analyses without AQ and SDQ.

For the first transition analysis, we performed a hierarchical logistic regression analysis for the children who were in a regular school in one wave and might have transitioned to a special school in the next. In Model 1, we entered the child- level variables: gender, socioeconomic status, intelligence and co- occurring conditions. The likelihood of a transition from a regular to a special school was higher for children with lower intelligence (*p* < 0.05). No other predictor at the level of the child was related to the likelihood of transition. In model 2, we added age of the child. The likelihood of a transition was higher for younger children (*p* < 0.05). In model 3, we added year (linear and quadratic trend) as predictors. The likelihood of a transition was higher for the early waves than for the later (linear trend, *p* < 0.05). The findings for age and child- level variables did not change, see Table 5.

**Table 5:** Results for the transition analysis from a regular to a special school

Predictors	Model 1		Model 2		Model 3	
	Estimate	SE	Estimate	SE	Estimate	SE
Gender^a^	− .70	.31	.00	.31	.11	.32
Socioeconomic status	− .05	.08	− .08	.09	− .06	.09
Intelligence	− .44**	.16	− .43**	.16	− .44**	.16
Co-occurring conditions	.16	.28	.11	.28	.18	.28
Age of the child			− .18***	.05	− .19***	.06
Year					− .26***	.07
Quadratic trend of year					.06	.04
Constant	.49	1.06	2.81*	1.26	2.23	1.30
*Chi*^*2*^	(*Df* = 4) 9.28		(*Df* = 5) 21.30***		(*Df* = 7) 37.37***	

*p < 0.05, **p < 0.01, ***p < 0.001

^a^Gender coded as 1: male; 2: female

A second transition analysis was performed for children who started in special education and could thus remain there or transition to regular education in the next wave. The likelihood of a school transition from a special to a regular school was in each model higher for children with higher intelligence (*p* < 0.05) and older children (*p* < 0.05). These findings are the mirror image of the findings of the transition analysis with the transition from a regular to a special school, where younger children and those with lower intelligence were more likely to transfer schools. In this analysis year was not related to a transition, see Table 6.

**Table 6:** Results for the transition analysis from a special to a regular school

Predictors	Model 1		Model 2		Model 3	
	Estimate	SE	Estimate	SE	Estimate	SE
Gender^a^	.58	.38	.61	.38	.59	.39
Socioeconomic status	− .12	.10	− .12	.10	− .12	.10
Intelligence	.41**	.15	.39^**^	.14	.39^**^	.14
Co-occurring conditions	− .22	.33	− .25	.33	− .25	.33
Age of the child			.20^**^	.08	.21^**^	.08
Year					.04	.09
Quadratic trend of year					.03	.05
Constant	− 4.66***	1.23	− 7.19***	1.59	− 7.29^***^	1.61
*Chi*^*2*^	(*Df* = 4) 12.38*		(*Df* = 5) 20.14**		(*Df* = 7) 21.19**	

*p < 0.05, **p < 0.01, ***p < 0.001

^a^Gender coded as 1: male; 2: female

In both transition analysis, none of the planned interaction terms (interaction between year and educational setting, three -way interaction between year, educational setting and age, and a three- way interaction between educational setting, age and gender) reached significance.

**Fig. 2:**
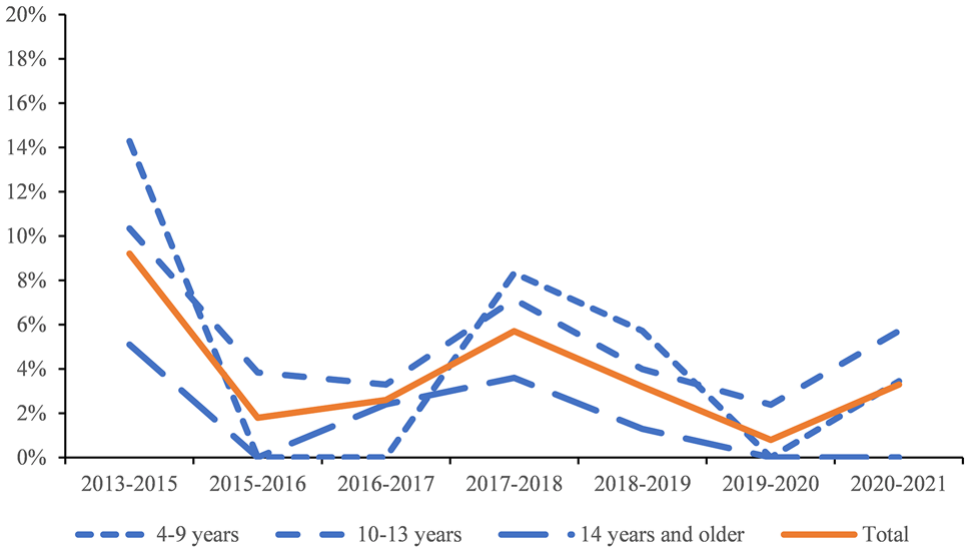
Likelihood of transition from a regular to a special school over the years for each age category

To visualize the transitions between regular and special schools as a function of age of the child and of wave, we grouped children in age groups of approximately equal size, of 4–9 years (203 children), 10–13 (611 children) and 14 and older (549 children). Figure 2 shows that the likelihood of a transition from a regular to a special school was especially large for younger children in the interval between 2013 and 2015, in which the AEA was introduced.

**Fig. 3:**
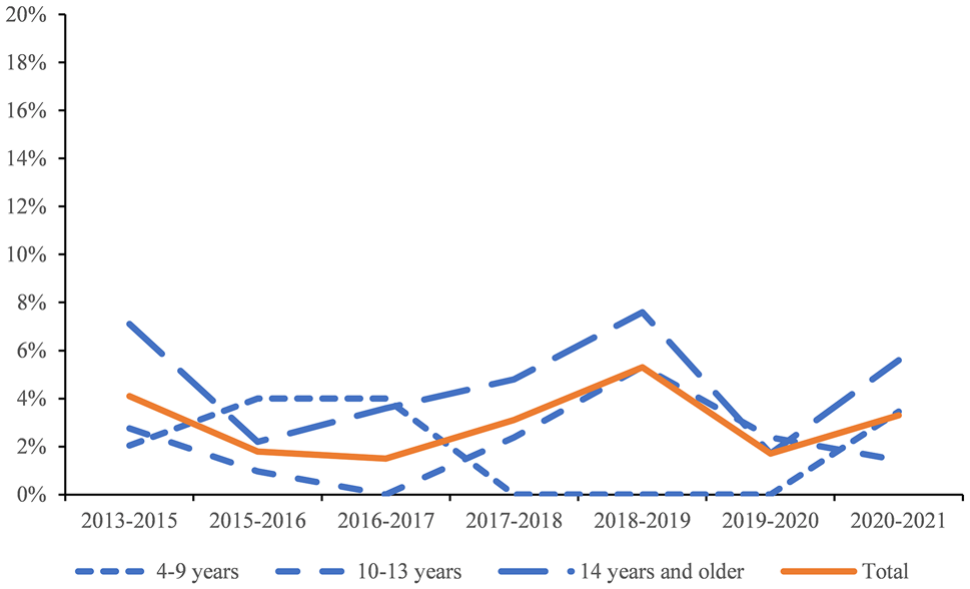
Likelihood of transition from a special to a regular school over the years for each age category

Figure 3 visualizes the likelihood of reverse transitions from a special school to a regular school over the years for each age category.

Here no effect of year was found, but older children were more likely to make this transition than younger.

## Discussion

The current study explored whether the introduction of a nationwide inclusive policy (Appropriate Education Act, AEA) was associated with regular or special school placement of children with autism aged 5 to 16 years. Furthermore, the impact of individual characteristics on transitions was investigated.

Surprisingly, the implementation of the inclusive policy act did not lead to a decrease in the proportion of autistic children placed in special schools. The proportion of special school placements even slightly increased. Autistic children with lower intelligence scores, co- occurring conditions, higher scores on behavioral indicators (behavioral problems and peer relation problems), and boys were more likely to be placed in special schools, particularly if they had higher scores for behavioral problems and peer relation problems. Interestingly, in the initial years following the policy change there was a greater probability of students transitioning from regular schools to special schools, compared to the subsequent years. The likelihood of a transition from a special to regular school did not change in the subsequent years. Younger autistic children more often transferred from a regular to a special school, whereas a transition from special to regular was more likely for older children with autism. In addition, autistic children with lower intelligence scores were more likely to transfer from a regular to special school while those with higher intelligence scores were more likely to transfer from special to regular.

The limited AEA impact on special school placements is in line with the results in the AEA evaluation report (De Boer, 2020; Ledoux et al., 2020), and may be explained by the lack of commanding legislative mandates and clear pathways in the AEA for families of autistic children to press for compliance. Countries with a policy including such legislation, reported after the implementation of the policy change a substantial increase in the enrolment of children with autism in regular schools. Conversely, and in line with our findings, if the policy did not have such a legislation, no increase in regular school placement was reported (De Bruin, 2019; Gindi, 2020). Future research should focus on clarifying the school placement in association with the presence or absence of such legislations in the specific educational context of countries (Meijer & Watkins, 2019).

In our study, the majority of the transitions occurred in the initial years following the implementation of the policy change. The increases immediately after the policy change aligns with findings of De Bruin (2019) which reported a 41% increase of in special schools placements for autistic children in the first decade following the implementation of the inclusive policy in Australia. This pattern may be explained by an accelerating effect of the new policy. School staff might have expected that access to the special education services would be restricted in the years after introduction of the policy, and therefore have already referred children who were struggling at a regular school to a special school.

In the current study, younger children transferred more often from a regular to special schools, while older children were more likely to transition from special to regular. Autistic children seem to make the transition from special to regular at the end of the primary school period (6th and 7th grade); no evidence was found for an earlier or later transfer to a special school since the AEA. Our findings suggest that children with autism were mostly transferred at the natural transition point from primary to secondary education. The decision to transfer may have been postponed to the end of the primary school period to avoid anxiety and increased social demands. Parents and school staff may also believe that changing an educational environment is accompanied by uncertainty related to support services and specialized educators (Brede et al., 2017; Nuske et al., 2019). Our findings are in accordance with Gindi (2020) and White et al. (2007) who found that children with autism often remain in their initial educational setting in which they are placed. This highlights the need, as emphasized by Lord et al. (2022), for professionals in education to be more alert to the potential for change in the need of children with autism in order to promote their accessibility to education.

As expected and consistent with previous studies (Lauderdale-Littin et al., 2013; Rattaz et al., 2020; Sansosti & Sansosti, 2012; Yianni-Coudurier et al., 2008), behavioral problems and peer relation problems were positively related to autistic children’s special school placement. In accordance with Roberts and Simpson (2016), behavioral indicators that are perceived as disruptive by the environment may lead to special school placement. In regular education settings, such behaviors might pose concerns by school staff about the safety and the realization of the academic achievement of the other children in their class and might be viewed as inappropriate for a regular school. Understanding and managing challenging behavior were mentioned as barriers to the inclusion of children with autism (Lindsay et al., 2013; Stephenson et al., 2021). Attention/ hyperactivity nor emotional problems were related to special school placement. Emotional problems, representing internalizing anxiety- related behavioral indicators, were associated with placement in a regular school. Such problems often are the result of a less adapted educational environment at regular schools having e.g., larger classes and less autism specific teachers’ knowledge (Green, 2019).

The present study has several limitations. No data was available prior to 2013, when the AEA was introduced. There was a significant amount of missing data for the behavioral indicators and autistic traits which reduced the power to detect their contribution to school placement. Furthermore, the sample size showed a declining trend over time. This probably happened because the 2013 wave participants were recruited from the entire Dutch Autism Society membership, which comprised around 14,000 members, leading to a high initial enrollment. In subsequent we saw a decline in participation, due to the follow up measures which required pseudo anonymity. Subsequent waves consisted of continuing participants, and new recruits, who were fewer in number.

In the current study the SDQ measuring the behavioral indicators were collected over a relatively short period of time (2017 and 2018) and only the most recent scores were used. Therefore, it is unknown whether the attention/hyperactivity and emotional problems were as prominent at the time of school placement or transition. Behavioral indicators in children with autism may evolve over time (Simonoff et al., 2013). By using the most recent SDQ scores, the impact of evolving behavioral indicators on school placement could be underestimated.

It is worth noting that the category special school in the Netherlands includes a variety of special schools, such as special schools for children with psychiatric disorders and behavioral problems, intellectual disability, or language-speech development disorders. Given the heterogeneity of autism (Lord et al., 2022), it is possible that some children in our sample are mainly placed in a special school because of a co- occurring condition rather than their autism. The contribution of co-occurring conditions should be considered when results are interpreted.

Finally, the number of transitions is small. In addition, the proportion of girls with autism-who are generally underrepresented in autism research- in our sample is relatively small, about 20% to 25% in each wave, making it difficult to draw firm conclusions.

Combining school attendance over time and child characteristics, our results suggest that the inclusive policy had no significant impact on the school placement in the Netherlands. The policy may have only resulted in a delay in referrals to special schools, which is contradictory to the policy’s goals. Our study is one of a few studies focusing on the school placement and transitions of children with autism in inclusive education and concluded that inclusive education has not yet been successfully implemented. As in other countries, the educational system of the Netherlands is moving towards inclusive education (Van Kessel et al., 2021), but the legislative mandates should be more commanding and pathways for families should be clarified to ensure that every child with autism is placed in a school where it is able to benefit from high-quality learning and experience pleasure in their school attendance.

The current study underscores practical considerations. Increased knowledge about autism and more awareness of the interaction of the autistic child with their school environment could lead to a better understanding of the evolving educational support needs of autistic children throughout life. The inclusive policy could promote structural support network to provide knowledge exchange between special and regular schools (Roberts & Simpson, 2016; Van Kessel et al., 2021). During the annual evaluation of the autistic child’s development, teachers and stakeholders should critically analyze which educational setting is most appropriate. The findings of the current study stress the importance of adjustments in the inclusive policy to ensure for successfully inclusion of autistic children in education.

## Supplementary Information

Below is the link to the electronic supplementary material.

Supplementary file1 (PDF 229 kb)

## Data Availability

The data for this research is available on request.

## Code Availability

The software code for this research is not available.

## Declarations

### Research Involving in Human and Animal Participants

All procedures performed in this study involving human participants were in accordance with the ethical standards of the institutional and/or national research committee.
