# Supplementary material for: Longitudinal Transition Between Regular and Special Education in Autistic Children: Predictors and Policy Effects
Source: J Autism Dev Disord. 2024 May 20;55(8):2651–62. doi: 10.1007/s10803-024-06369-4 (PMC12296867; doi:10.1007/s10803-024-06369-4)
Supplement: Supplementary file 1 — Supplementary file1 (PDF 229 kb) [file 10803_2024_6369_MOESM1_ESM.pdf]

Supplemental Material

Missing values

Over time, the sample size decreased and therefore there are missing datapoints for autistic traits (AQ-short), behavioral indicators (SDQ) and family social economic status, see Table S1.

Table S1

Number of Measurements for the Predictor Variables in 2013 to 2021

Wave	Gender	Social economic Status	Intelligence	Autistic traits	Behavioral indicators	Co- occurring conditions	Age of the child
2013	847	432	842	363	238	842	847
2015	555	555	553	460	267	539	555
2016	331	295	330	232	223	318	331
2017	399	367	394	272	347	382	399
2018	379	353	338	270	285	360	379
2019	332	318	301	243	183	316	332
2020	312	298	284	229	98	297	312
2021	230	226	207	166	78	220	230
N	3385	2844	3249	2235	1719	3256	3385

To assess the implications of the small samples due to the missing values for autistic traits and behavioral indicators, we repeated our transition analysis with autistic traits and behavioral indicators to assess the contribution of the year in the complete sample. Compared to the first main analysis, the sample decreased from 574 to 359. In each model of this analysis, the likelihood for a transition was higher for the early waves than for the later (linear trend, (B= -.29 = .75, SE=.13, p = .020). In none of the models, the quadratic trend of year

was related to the likelihood for a school transition from a regular to a special school (B =.08, SE = 08, *p* = .263). Autistic traits (B=.01, SE = 01, p = .442) and behavioral indicators (B= .01, SE = .03, p = .855) did not reach significance in all models.

We also repeated our second analysis for children who started in special education, and could thus remain there or transition to regular education in the next wave. Compared to the main analysis, the sample decreased from 935 to 444. Similar to the main analysis, in none of the models year was related to a transition from a special to a regular school (B=.05, SE= .18, p = .767). Neither was its quadratic trend (B=-.02, SE = .08, p = .830). Autistic traits reached significance from model 4 (B =-.05, SE= .02, p = .047), behavioral indicators did not reach significance in any of the models.

Comparison including special primary school as regular school

To allow better comparison of our results with countries that do not have something akin to special primary education, we also performed a placement analysis and a transition analysis with special school versus regular schools incl. special primary schools as the dependent variable. These analyses are additional to the ones reported in the main text, with special school (including special primary school) versus regular school as the dependent variable.

Just like in the main placement analysis, the likelihood of placement in a special school in each model was higher for males, lower intelligence and the presence of co-occurring conditions (p <.05). Different from the main analysis, the likelihood for a placement in a special school was higher for older children and for the early waves (p <.05). Unlike the main placement analysis, behavioral indicators were not related to the likelihood of placement in a special school, see Table S2.

Table S2

The Results for the Placement Analysis with SO versus Regular incl. Special Primary School as Dependent Variable

Predictors	Model 1		Model 2		Model 3		Model 4	
	Estimate	SE	Estimate	SE	Estimate	SE	Estimate	SE
Gender[a]	-.73***	.17	-.79***	.17	-.79***	.17	-.80***	.17
Socioeconomic Status	-.02	.04	-.02	.04	-.02	.04	-.02	.04
Intelligence	-.30***	.07	-.33***	.08	-.33***	.08	-.34***	.08
Autistic traits	-.01	.01	-.01	.01	-.01	.01	-.01	.01
Behavioral indicators	.01	.01	.02	.01	.02	.01	.02	.01
Co- occurring conditions	.70***	.14	.70***	.14	.70***	.14	.70***	.14
Age of the child			.12***	.03	.12***	.03	.14***	.03
Inclusion Policy					-.06	.21	.43	.31
Year							-.13*	.06
Quadratic trend of year							.04	.02
Constant	1.85*	.76	.52	.82	.52	.82	-.24	.90
Chi²	(*Df*=6) 70.10***		(*Df*=7) 90.11***		(*Df*=8) 90.20***		(*Df*=10) 94.94***	

Notes * (p<0.05) ** (p<0.01) *** (p<0.001) [a] *Gender coded as 1: male; 2: female.*

Just like in the main analyses, we performed an additional placement analysis with the SDQ subscales following behavioral indicator scores: emotional problems, behavioral problems, hyperactivity/ inattention and peer problems. As shown in Table S3, peer problems were associated with higher likelihood of placement in a special school in each model ($p < .05$). Unlike the main placement analysis with SDQ- subscales, in this analysis behavioral problems were not associated with the likelihood of placement in a special school. In none of the models emotional problems and hyperactivity/inattention were related to the likelihood of placement in a special school. This did not differ from the main placement analysis with SDQ-subscales.

Table S3

The Results for the Placement Analysis with the Subscales SDQ and SO versus Regular incl. Special Primary School as Dependent Variable

Predictors	Model 1		Model 2		Model 3		Model 4	
	Estimate	SE	Estimate	SE	Estimate	SE	Estimate	SE
Gender[a]	-.63***	.18	-.67***	.18	-.67***	.18	-.68***	.18
Socioeconomic Status	-.02	.04	-.02	.04	-.02	.04	-.02	.04
Intelligence	-.28***	.08	-.30***	.08	-.30***	.08	-.31***	.08
Autistic traits	-.01	.01	-.01	.01	-.01	.01	-.01	.01
Emotional problems	-.05	.03	-.05	.03	-.05	.03	-.05	.03
Behavioral problems	.04	.04	.04	.04	.04	.04	.04	.04
Hyperactivity/ inattention	.01	.03	.02	.03	.02	.03	.03	.03
Peer problems	.09**	.03	.09**	.03	.09**	.03	.08**	.03
Co- occurring conditions	.68***	.14	.67***	.14	.67***	.14	.67***	.14

Notes * (p<0.05) ** (p<0.01) *** (p<0.001) [a] *Gender coded as 1: male; 2: female.*

Table continues on the next page

Table S3 continued

Predictors	Model 1		Model 2		Model 3		Model 4	
	Estimate	SE	Estimate	SE		Estimate	SE	Estimate
Age of the child			.11***	.03	.12***	.03	.14***	.03
Inclusive education Policy					-.02	.21	.44	.31
Year							-.12*	.06
Quadratic trend of year							.04	.02
Constant	1.54	.80	.15	.87	.15	.87	-.61	.95
Chi²	(*Df*=9) 82.10***		(*Df*=10) 100.66***		(*Df*=11) 100.67***		(*Df*=13) 104.90***	

Notes * (p<0.05) ** (p<0.01) *** (p<0.001)

We again performed the analysis without autistic traits and behavioral indicators, were there was a loss of datapoints due to missing values. This increased the sample from 1085 to 2367. Unlike in the main placement analysis without these two variables, in this analysis, the inclusive policy was not related to the likelihood of placement in a special school (B=.37, SE =.20, p = .064). The likelihood for placement in a special schools did decrease with year of wave ($p < .05$), see Table S4, which is different from the main placement analysis in which year was not related to the likelihood of placement in a special school when autistic traits and behavioral values were excluded from the analysis.

Table S4

The Results for the Placement Analysis without AQ and SDQ and with SO versus Regular incl. Special Primary School as Dependent Variable

Predictors	Model 1		Model 2		Model 3		Model 4	
	Estimate	SE	Estimate	SE	Estimate	SE	Estimate	SE
Gender[a]	-.63***	.11	-.65***	.11	-.65***	.11	-.65***	.11
Socioeconomic Status	-.01	.03	-.00	.03	-.00	.03	.00	.03
Intelligence	-.61***	.04	-.62***	.04	-.62***	.04	-.63***	.04
Co- occurring conditions	.35***	.09	.34***	.09	.34***	.09	.34***	.09
Age of the child			.09***	.02	.09***	.02	.09***	.02
Inclusion Policy					-.02	.12	.37	.20
Year							-.10*	.04
Quadratic trend of year							.02	.01
Constant	3.38***	.34	2.37***	.38	2.37***	.39	1.93***	.43
Chi²	(*Df*=4) 312.11***		(*Df*=5) 342.72***		(*Df*=6) 342.74***		(*Df*=8) 349.33***	

Notes * (p<0.05) ** (p<0.01) *** (p<0.001) [a] *Gender coded as 1: male; 2: female.*

Also the transition analysis was performed for this different grouping of schools. Unlike in the main placement analysis, the likelihood for a transition from a regular to a special school was not related to intelligence scores. The other variables at the level of the child were also not related to a transition. In model 2, then we added age of the child as a prediction to the model. Unlike, the original transition analysis, age of the child was not related to the likelihood for a school transition from a special to a regular school. In model 3, we added year and its quadratic trend to the model. Just like in the main analysis, the likelihood for a transition was higher for the early waves ($p < .05$) but the quadratic trend of year was not related to a transition, see Table S5.

Table S5

Results for the Transition Analysis from a Regular to a Special School with SO versus Regular incl. Special Primary School as Dependent Variable

Predictors	Model 1		Model 2		Model 3	
	Estimate	SE	Estimate	SE	Estimate	SE
Gender[a]	-.31	.37	-.30	.37	-.23	.37
Socioeconomic Status	.01	.09	.01	.09	.03	.09
Intelligence	-.23	.15	-.22	.15	-.24	.16
Co- occurring conditions	-.31	.32	-.32	.32	-.29	.32
Age of the child			-.02	.06	-.02	.06
Year					-.33***	.08
Quadratic trend of year					.07	.05
Constant	-.62	1.15	-.34	1.34	-1.06	1.38
Chi²	(*Df*=4) 3.68		(*Df*=5) 3.85		(*Df*=7) 23.48 **	

Notes * (p<0.05) ** (p<0.01) *** (p<0.001) [a] *Gender coded as 1: male; 2: female.*

This finding is different from the main analysis, in which the quadratic trend was not related to the likelihood of a transition.

Just like in the main transition analysis, we also analyzed transitions for children who were in a special school, and might remain there or transition to a regular school. First, we entered the child- level variables gender, social economic status, intelligence and co-occurring conditions. Just like in the main analysis of such transitions, the likelihood of a school transition from a special school to a regular school was higher for children with higher intelligence ($p < .05$). Only in the first model, the likelihood for a transition from special to regular school was higher for females. The other variable at the level of the child were not predicting school transition. In model 2, then we added age of the child as a predictor to the model. Unlike the main analysis of special-to-regular transitions, age of the child was not related to the likelihood of a school change. In model 3, we added year (linear and quadratic trend) as predictors to the model. Both, year and its quadratic trend were not related to the likelihood of a school change from a special to a regular school , see Table S6.

Table S6

Results for the Transition Analysis from a Special to a Regular School with SO versus Regular incl. Special Primary School as Dependent Variable

Predictors	Model 1		Model 2		Model 3	
	Estimate	SE	Estimate	SE	Estimate	SE
Gender[a]	.80*	.39	.76	.39	.73	.39
Socioeconomic Status	-.13	.10	-.12	.10	-.13	.10
Intelligence	.42**	.14	.40**	.14	.40**	.14
Co- occurring conditions	-.22	.33	-.23	.33	-.23	.33
Age of the child			.13	.08	.13	.08
Year					.05	.09
Quadratic trend of year					.02	.05
Constant	-4.80***	1.25	-6.28***	1.56	-6.33***	1.58
Chi²	(*Df*=4) 14.70**		(*Df*=5) 17.66**		(*Df*=7) 18.57*	

Notes * (p<0.05) ** (p<0.01) *** (p<0.001) [a] *Gender coded as 1: male; 2: female.*

Just like in both of the main transition analysis, none of the planned interaction terms (interaction between year and educational setting, three -way interaction between year, educational setting and age, and a three- way interaction between educational setting, age and gender) reached significance.
